# Books and Magazines

**Published:** 1914-04

**Authors:** 


### Books and Magazines.

The Strange Case of Dr. Bruno. By F. E. Daniel, M. D. Cloth. 235 pages. Price, $1.50. Austin, Texas: Von Boeckmann-Jones Company, 1913.

The gifted editor of *The Texas Medical Journal* has woven an interesting tale about a young physician, a blending of romance, love, scientific fact, philosophy and imagination, in which suspended animation by means of a synthetic drug plays an important part. It is a strangely fascinating story, weird in its realities, awakening in the reader every impulse which is governed by his inner consciousness. It is one of those stories which cannot be told. It must be read in its entirety.—*Medical Times* (N. Y.)

---

A REFERENCE HAND-BOOK OF THE MEDICAL SCIENCES. Embracing the entire range of scientific and practical medicine and allietd science. By various writers. Third edition. Completely revised and rewritten. Edited by Thomas Lathrop Stedman, A. M., M. D. Complete in eight imperial quarto volumes. Volume III. 934 double-column pages, illustrated by 659 engravings and seven full-page plates in colors. Wm. Wood & Co., New York.

The third edition of the "Reference Hand-book," which has been called "the most popular medical book ever published," bids fair to gain even greater popularity than was enjoyed by the first and second editions. The third volume, just issued, is, if anything, superior to Volumes I and II. Of the 539 individual articles contained in Volume III, it is manifestly impossible to mention more than a few, but several of these are conspicuous for their especial excellence, such as: Dislocations; embryological articles; criminology; asexualization of criminals and defectives; cranial nerves; surgery of the colon; colon bacillus infections; diseases of the eye; Huntington's chorea; color perception and tests for color blindness; diabetes; electrocoagulation; electrodiagnosis; diathermy; dysentery; dermatitis; disinfection; articles on materia medica, health resorts and mineral springs; biographies of physicians of ancient and modern times. A great number of very short definitions of terms more exhaustively treated in other articles, is a feature of great usefulness. In mechanical make-up the book would be hard to excel. The paper is excellent, the illustrations of a high grade, both the cuts in the text and the colored plates.

